# Thermostable, Dissolvable Buccal Film Rotavirus Vaccine Is Highly Effective in Neonatal Gnotobiotic Pig Challenge Model

**DOI:** 10.3390/vaccines9050437

**Published:** 2021-04-30

**Authors:** Casey Hensley, Peng Zhou, Sofia Schnur, Hassan M. Mahsoub, Yu Liang, Min-Xuan Wang, Caroline Page, Lijuan Yuan, Victor Bronshtein

**Affiliations:** 1 Department of Biomedical Sciences and Pathobiology, Virginia-Maryland College of Veterinary Medicine, Virginia Tech, Blacksburg, VA 24061, USA; lhcasey@vt.edu (C.H.); pengz81@vt.edu (P.Z.); sschnur@vt.edu (S.S.); hmahsoub@vt.edu (H.M.M.); yul20@vt.edu (Y.L.); 2 Universal Stabilization Technologies, Inc., San Diego, CA 92121, USA; minw@vitrilife.com (M.-X.W.); carolinep@vitrilife.com (C.P.)

**Keywords:** thermostable, oral film vaccine, rotavirus, diarrhea, gnotobiotic pigs

## Abstract

Difficulties related to storage and transport of currently available live oral rotavirus vaccines can have detrimental consequences on the efficacy of the vaccines. Thus, there is a great need for thermostable vaccines that can eliminate the necessity for cold chain storage or reconstitution before administration. In this study, we developed a dissolvable oral polymeric film comprised of a live attenuated thermostable tetravalent rhesus-human reassortant rotavirus vaccine (RRV-TV) powder and antacid (CaCO_3_). Immunogenicity and protective efficacy of the vaccine after buccal delivery was evaluated in the gnotobiotic pig model of human rotavirus (HRV) infection and diarrhea. Two doses of the vaccine were highly immunogenic and conferred strong protection against virus shedding and diarrhea upon challenge with a high dose of a virulent G1 HRV in gnotobiotic pigs. Those pigs vaccinated with the preserved film vaccine had significantly delayed onset of diarrhea; reduced duration and area under the curve of diarrhea; delayed onset of fecal virus shedding; and reduced duration and peak of fecal virus shedding titers compared to pigs in both the placebo and the reconstituted liquid oral RRV-TV vaccine groups. Associated with the strong protection, high titers of serum virus neutralization antibodies against each of the four RRV-TV mono-reassortants and G1 HRV-specific serum IgA and IgG antibodies, as well as intestinal IgA antibodies, were induced by the preserved film vaccine. These results demonstrated the effectiveness of our thermostable buccal film rotavirus vaccine and warrant further investigation into the promise of the novel technology in addressing drawbacks of the current live oral HRV vaccines.

## 1. Introduction

Rotavirus is the leading cause of severe infantile diarrhea worldwide. Even with live oral vaccines widely available since 2006, rotavirus infections are still responsible for the majority of cases of diarrheal disease in children globally, accounting for up to 50% of these cases [1,2]. Of the many children under five years of age infected with rotaviruses, up to 215,000 die each year from this vaccine-preventable disease, and 90% of these deaths occur in low- and middle-income areas of Asia and Africa [2,3,4].

Rotavirus vaccine development has been most successful in the area of live attenuated, orally delivered vaccines. There are currently two licensed human rotavirus (HRV) vaccines available on the markets in the United States and many other countries, though there are others in various stages of preclinical and clinical trials [5]. RotaTeq^®^ (Merck, Kenilworth, NJ, USA) and Rotarix^®^ (GSK, Brentford, UK) are both prequalified for worldwide use by the World Health Organization (WHO), and as of 2009, WHO has recommended that HRV vaccines be incorporated into all national immunization programs [6]. These vaccines are highly efficacious (~90%) at preventing severe rotaviral diarrhea in properly vaccinated children in the United States and other high-income countries, but their efficacies are much lower in low- to middle-income countries (LMIC) (39–70%) and the cause is multi-faceted [1,7,8,9,10,11,12,13].

Despite their high efficacy in developed countries, RotaTeq^®^ and Rotarix^®^ lack some important characteristics for maintaining their efficacy in developing countries and increasing their distribution worldwide. First, both vaccines require consistent and stable temperatures for storage and transport (2 °C to 8 °C) and are sensitive to extreme conditions. Fluctuations in temperature can lead to degradation of the vaccine components, resulting in inadequate dosing. Along with this, both vaccines are in either a liquid form (RotaTeq^®^) or freeze-dried powder requiring reconstitution with a liquid diluent (Rotarix^®^). This complicates the administration of vaccines to infants. According to the Vaccine Adverse Events Report System, the most common issues with both vaccines are related to the infant spitting up the liquid, diluent mix-ups, accidental injection, and accidental splashing into administrator’s eyes [14]. All of these scenarios present not only a health risk to healthcare workers, but also the risk of inadequate dosing and subsequent impaired protective efficacy for the infants receiving the vaccine. Furthermore, HRV vaccines are prohibitively expensive in LMIC, even in those receiving Gavi funding and subsidization [15,16]. A large portion of the costs for vaccines is their distribution that requires an intact cold chain. Based on an article published at The Conversation, WHO estimated that up to 50% of vaccines get wasted globally every year because of temperature control, logistics, and shipment-related issues [17]. The appeal of inexpensive, thermostable, and easily administered vaccines is clear.

The first objective of this study was to develop a thermostable rotavirus vaccine in a mucoadhesive dissolvable polymeric film for reconstitution-free administration to the buccal or sublingual surfaces in order to provide simpler, more accurate dosing to neonates and to allow storage, shipment, and delivery of the vaccine at ambient temperatures. The second objective was to evaluate the immunogenicity and protective efficacy of the buccally administered thermostable tetravalent human-rhesus rotavirus reassortant (RRV-TV) film vaccine in a neonatal gnotobiotic (Gn) pig model of HRV infection and disease.

RRV-TV was selected because it is a proven vaccine: it was first licensed in 1998 but withdrawn in 1999 due to a rare association with intussusception, which occurred disproportionately in infants receiving their first dose at more than three months of age [18]. A phase II clinical trial with infants receiving the first dose during the neonatal period (0–27 days) and the second dose before 60 days of age demonstrated its safety, immunogenicity and efficacy of 63.1% against rotavirus gastroenteritis of any severity associated with any of the four serotypes in the vaccine in Africa [19]. The vaccine is slated for commercialization again as a cost-effective alternative to Rotarix^®^ and RotaTeq^®^ for Gavi-eligible countries.

The Gn pig model is very well established, and is routinely used in HRV vaccine pre-clinical evaluation [20]. It is the only available model large enough for buccal administration of the full-size film designed for human infants. This model is not only ideal due to its size, but for its physiologic, anatomic, and immunologic similarities to human infants. Their similar development of antibodies, lymphoid organs and cell-mediated immunity make them a practical choice for translational vaccine research [21,22]. In this study, the duration and severity of diarrhea and virus shedding in Gn pigs were monitored to assess the protection conferred by the vaccine against challenge with a virulent HRV strain. The intestinal and systemic virus-specific IgA and IgG antibody and serum virus-neutralizing antibody responses to G1-G4 rotaviruses after vaccination and challenge were measured to assess the immunogenicity of the preserved vaccine.

Polymeric films comprising vaccines for oral delivery have been formulated before [23,24]. In both of these formulations, polymeric film was produced by solvent casting from aqueous solutions, which limits both the vaccine stability that can be achieved at high ambient temperatures and incorporation of the amount of antacid necessary to protect from low gastric pH during intestinal delivery. Universal Stabilization Technologies, Inc. (UST) has introduced an alternative approach for production of thermostable vaccines encapsulated in water-dissolvable polymeric films [25], which comprises two steps: (1) Formulation of thermostable vaccine powders using Preservation by Vaporization (PBV) [26] and subsequent micronization; (2) Anhydrous encapsulation of vaccine powder in polymeric films using polymers soluble in both acetone and water (Bronshteing 2019 Patent US 10,272,033 B2) [25]. This allows anhydrous production of films from an acetone-based polymer solution comprising suspensions of dry vaccine powders and other dry excipients/buffers (i.e., antacids) if needed.

PBV is a state-of-the-art foam-drying process that has been successfully used to produce various thermostable biologics, including live vaccines [27,28,29]. This technology is efficient, inexpensive, and drastically increases the shelf-life of its products without compromising the potency of the components [25,27,30]. PBV-preserved live attenuated rabies virus (RABV) did not lose viral titers or antigenic content when stored at ambient temperatures and was effective in inducing neutralizing antibodies and protection in mice from peripheral RVBV challenge [28]. A live attenuated influenza vaccine (LAIV) intranasal powder was also developed from PBV LAIV and shown to be thermostable, immunogenic, and protective in ferrets against homologous challenge [29].

Using UST’s approach, we formulated a thermostable PBV rotavirus vaccine and demonstrated stability of the vaccine encapsulated in polymeric films at ambient temperatures. This dissolvable oral film containing thermostable RRV-TV was designed for buccal delivery, thus mitigating the previously described disadvantages of currently used oral HRV vaccines.

## 2. Materials and Methods 

### 2.1. Vaccine Formulation

In this study, we used an RRV-TV vaccine containing three human-rhesus mono-reassortant rotaviruses carrying G1, G2, and G4 VP7 of HRV respectively, on the RRV MMU18006 strain backbone and the G3 RRV (P5B[3]G3) [31]. Bulk live attenuated rotavirus vaccines were sourced from BravoVax Co. Ltd. in Wuhan, China [18,19].

#### 2.1.1. Preservation by Vaporization

Rotavirus G1, G2, G3, G4 serotype vaccines were preserved individually using PBV [26]. Before drying, vaccines were mixed 1:1 with preservation solution comprising 35% sucrose and 5% mannitol. The vaccine-preservation solution mixture was distributed into 0.5 mL portions in 5 mL borosilicate glass serum vials, and dried using the PBV foam drying process in a conventional VirTis Genesis freeze-dryer (Virtis, Gardiner, NY, USA). PBV protocol consisted of less than 2 h of primary drying starting after the application of vacuum, after which the material became a stable foam, followed by secondary drying, which included 20 h at 45 °C under vacuum. The vials were then sealed under vacuum. Vials of PBV-dried rotavirus vaccine foams were stored in bags with Drierite desiccant at room temperature (RT; 22 °C ± 3 °C) and in incubators (37 °C) for use in film fabrication or subsequent long-term stability evaluation.

#### 2.1.2. Micronization

To produce thermostable vaccine powder, PBV-dried rotavirus vaccine foams that had been stored at RT for 12 months were micronized in a humidity-controlled dry room at 13–15% relative humidity (RH). Micronization into powder was achieved using a Laarmann Labwizz lab-scale ball mill and sieved through a 63 µm mesh.

#### 2.1.3. Film Fabrication

Dissolvable vaccine films were produced at UST using a conventional solvent casting approach. Films were designed for buccal administration to human infants. Each one square-inch film contained 60 mg of micronized PBV RRV-TV (4 × 10^5^ focus forming units (FFU)/dose) and 60 mg calcium carbonate (CaCO_3_) as antacid to protect viruses from low gastric pH during oral delivery. The dose of 60 mg of CaCO_3_ was selected based on similar antacid dosing contained in Rotarix^®^ during administration [32].

Micronized rotavirus vaccine and antacid (CaCO_3_) powders were mixed into an acetone solution comprising 8.5% *w*/*w* hydroxypropyl cellulose (HPC; M_w_ 80,000; Sigma Aldrich, St. Louis, MO, USA) and 0.75% *w*/*w* triacetin (plasticizer) to produce the polymeric vaccine mixture. HPC is soluble both in water and acetone and is a GRAS-listed ingredient. The polymeric vaccine mixture was poured onto a substrate sheet on an Elcometer 4340 automatic film applicator machine (Elcometer, Manchester, UK) and spread using a doctor blade at 3.0 mm thickness. The film sheet was air dried inside a humidity-controlled dry room (13–15% RH) for three hours, then was additionally dried under vacuum for one hour. The dry film sheet was removed from the substrate, weighed, and cut into dose-appropriate pieces. Film pieces were distributed into 10-mL serum vials and dried under vacuum to remove remaining acetone, and subsequently sealed under vacuum for use in stability and challenge studies.

### 2.2. Rotaviruses for Challenge and Immunoassays

The challenge virus inoculum used for this study was derived from a pool of intestinal contents from the 29th passage of the virulent Wa strain (G1P[8]) HRV (VirHRV) in Gn pigs. The median infectious dose (ID_50_) and median diarrhea dose (DD_50_) of the VirHRV in Gn pigs were previously determined to be approximately 1 FFU [33]. The lysates of Wa HRV-infected African green monkey kidney MA104 cells (ATCC CRL-2378.1™) were partially purified by centrifugation through 40% (wt/vol) sucrose cushion and used as detector antigen in the ELISA for the detection of serum and intestinal antibody responses in Gn pigs as described previously [34].The human-rhesus mono-reassortant rotavirus G1, G2, G4 strains and G3 RRV strain were used in the virus neutralization (VN) assays for the detection of G type specific serum VN titers in the Gn pigs.

### 2.3. Vaccine Inoculation and Virus Challenge of Gn Pigs

The vaccine films and PBV RRV-TV powders were vacuum sealed in serum vials and transported in one shipment on ice by overnight FedEx t from Universal Stabilization Technologies, San Diego, CA to Virginia Tech, where they were stored at 4 °C upon arrival. They were stored for 42 days before being placed in the Gn pig isolators for the first vaccination of Gn pigs. The vaccines stayed in the isolator port for one hour under a controlled room temperature of ~30–34 °C (first dose at 34 °C on post-inoculation day [PID] 0 and second dose at 30 °C on PID 10) during the sterilization procedure.

Gnotobiotic pigs used in this study were surgically derived and maintained as previously described [20]. Gn pigs (large white crossbreed) were fed ultra-high temperature treated (UHT) sterile whole cow’s milk (The Hershey Company, Hershey, PA, USA) until PID 21 and were then transitioned to Similac^®^ baby formula (Abbott Laboratories, Chicago, IL, USA) until the end of the study. The pigs remained bacteria-free throughout the experiment confirmed by weekly rectal swabs tested on blood agar plates and in thioglycolate broth.

A total of 17 Gn pigs (both male and female) were assigned randomly to three groups: (1) Preserved film (*n* = 5); (2) Placebo film (*n* = 6); and (3) Liquid vaccine (*n* = 6). Pigs received the first dose of vaccine or control at five days of age (PID 0) and the second dose at 15 days of age (PID 10).

The preserved film vaccine and placebo film were administered buccally and the liquid vaccine was administered orally. The films in two 1 × 1 inch pieces were placed in the left and right cheek pouch of the pigs. Contact time was longer than anticipated, with time to dissolve taking up to 20 min. A PBV thermostabilized powder RRV-TV containing the 4 × 10^5^ FFU/dose was reconstituted in 5 mL of Diluent #5 [minimum essential media (MEM, ThermoFisher Scientific, Inc. Waltham, MA, USA); 100 IU of penicillin per mL, 0.1 mg of dihydrostreptomycin per mL; and 1% HEPES] immediately before use and a total volume of 5 mL was administered orally to Gn pigs for comparison of immunogenicity and protective efficacy with the preserved film vaccine. The liquid vaccine was fed through a needleless syringe and immediately swallowed by the pigs, with very brief contact to the buccal mucosa.

Serum samples were collected at PID 0, PID 10, PID 17, PID 28 and PID 35 for the detection of Wa HRV-specific IgA and IgG antibody responses by ELISA and G1, G2, G3, and G4 reassortant rotavirus strain-specific VN antibody titers by microplate VN assays. Pigs were orally challenged with 6 × 10^5^ FFU of VirHRV Wa strain at PID 28. Twenty minutes prior to vaccination and oral challenge with VirHRV, pigs were fed 4 mL of 200 mM sodium bicarbonate (NaHCO_3_) to neutralize stomach acidity. Clinical signs were monitored daily and rectal swabs were taken for the detection of virus shedding from postchallenge day (PCD) 0 to PCD 7.

All pigs were euthanized on PCD 7. At euthanasia, small and large intestinal contents (SIC and LIC) were collected aseptically and processed as previously described, for the detection of intestinal antibody responses and antigen presence by ELISAs, as well as infectious virion counting by cell culture immunofluorescence (CCIF) [35].

### 2.4. Assessment of Diarrhea and Detection of Fecal HRV Shedding by Rotavirus Antigen ELISA and CCIF

For the assessment of severity of diarrhea postchallenge, fecal consistency scores were recorded daily as 0: normal, 1: pasty, 2: semi-liquid, and 3: liquid [33,36]. Pigs are considered diarrheic with scores of ≥2. Daily rectal swabs were collected and used for HRV antigen detection by ELISA and infectious virus particle detection by CCIF as we described previously [34]. For using the capture ELISA to detect rotavirus antigen, a 96-well plate was coated with 100 µL/well goat anti-bovine rotavirus polyclonal unconjugated antibody (PA1-7241, ThermoFisher Scientific, Waltham, MA, USA; AB_561090) at a dilution of 1:250 in carbonate buffer (pH 9.6). The plate was incubated overnight at 4 °C, washed twice with PBST (PBS containing 0.05% Tween^®^ 20 (Sigma Aldrich, Co. St. Louis, MO, USA), blocked with 300 µL/well of PBS (pH 7.4) containing 5% nonfat dry milk then incubated for 1 h at 37 °C. After washing three times with PBST), 100 µL/well of diluted sample (in Diluent #5) was added in duplicate. Semi-purified AttHRV antigen or swab samples from HRV-negative Gn pigs were used as a positive and negative control respectively. Plates were incubated for 1 h at 37 °C then washed three times with PBST. Goat anti-bovine rotavirus polyclonal HRP-conjugated antibody (PA1-73015, ThermoFisher Scientific Waltham, MA, USA; AB_1018382) diluted 1:200 in PBS containing 1% BSA was then added to each well (100 µL/well) and incubated for 1 h at 37 °C, followed by three washes with PBST. The plate was developed with ABST peroxidase substrate solution for 15–30 min at room temperature before being stopped with ABST stop solution diluted 1:5. The optical density was measured at 405 nm using a microtiter plate reader [34].

### 2.5. Detection of Wa HRV-Specific Serum and Intestinal IgA and IgG Antibody by ELISA

To determine the Wa HRV-specific IgA and IgG antibody titers in serum, SIC and LIC samples, direct ELISA was performed as described previously, with modification [34]. Briefly, 96-well microtiter plates were coated with semi-purified attenuated Wa HRV antigen at 68 µg/mL or mock-infected MA104 cell culture control lysate at 68 µg/mL in 0.05 M carbonate buffer (pH 9.6) for 1 h at 37 °C. The concentration of the semi-purified attenuated Wa HRV antigen was determined by NanoDrop™ One (Thermo Scientific, Waltham, MA, USA) and titrated in the range of 68 to 102 µg/mL, with 68 µg/mL determined as the optimal coating concentration. Following the incubation, plates were washed five times with TBST (Tris-buffered saline with 0.05% Tween^®^ 20, pH 8.0) and blocked overnight at 4 °C with TBS‑5% BSA. In separate deep 96-well plates, samples were diluted 4-fold serially with TBST‑5% BSA, including one positive and two negative controls. One hundred µL/well of the serially diluted samples were then transferred to the coated plates and incubated overnight at 4 °C. The plates were washed five times with TBST and incubated in the dark at room temperature for 2 h with 100 µL/well horseradish peroxidase (HRP) conjugated goat anti-pig IgA antibody or HRP-conjugated goat anti-pig IgG-Fc antibody (Bethyl Laboratories, Inc., Montgomery, TX, USA) diluted 1:3000 in TBST-1% BSA. Plates were washed five times with TBST and developed with 100 µL/well room temperature ABTS peroxidase substrate (1:1 ratio of KPL ABTS^®^ peroxidase substrate solutions A and B from SeraCare Life Sciences, Inc., Milford, MA, USA) in the dark, for 15‑30 min before ABTS peroxidase stop solution was added. Optical density was read at 405 nm.

### 2.6. Detection of G1, G2, G3 and G4 Rotavirus Strain-Specific Serum VN Antibody Responses

Rotavirus G1 to G4-type specific VN antibody titers in sera of all pigs collected on PID 28 and PCD 7 were determined by a microplate cell culture immunofluorescence staining assay as described previously [34]. In brief, MA104 cells (passage 10 to 16) were grown in 96-well plates for three days to 100% confluence. Monolayers were washed once with Eagle’s minimum essential medium (EMEM) (Corning, NY, USA), and the 96-well plates were incubated with serum-free EMEM at 37 °C for 2 h. In a separate 96-well plate, serum samples were diluted 4-fold in duplicate from 1:4 to 1:16384 and then were incubated with 4 × 10^3^ FFU of mono-reassortant rotavirus G1, G2, G4 or RRV G3 strain for 1 h. The serum-free EMEM was discarded from the MA104 cell plates after a 2 h incubation. The plates were then inoculated in duplication with 50 µL/well of the mixtures of serum samples and G1 to G4 rotaviruses or EMEM and incubated at 37 °C for 1 h. Fifty µL of EMEM with trypsin (0.5 μg/mL) was added to each well and the plates were incubated at 37 °C with 5% CO_2_ for additional 18–24 h. The plates were fixed with 80% cold acetone and stained with goat anti-bovine rotavirus polyclonal antibody (PA1-7241, ThermoFisher Scientific, Waltham, MA, USA) and FITC-conjugated rabbit anti-goat IgG antibody (F7367, Sigma, Inc., St. Louis, MO, USA). Fluorescing cells were observed by fluorescent microscopy. The virus-neutralizing antibody titer was defined as the reciprocal of the maximum dilution of sera that began to have any fluorescing cells.

### 2.7. Vaccine Stability Assessment by Focus Forming Assay

PBV foams of individual rotavirus serotype vaccines and RRV-TV vaccine films were stored with Drierite desiccant for stability assessment at room temperature (RT; 22 °C ± 3 °C) and in incubators (37 °C). Samples were removed from storage and tested at specific time points. PBV foams were tested at initial yield (0 month), three months, six months, and 12 months. PBV foam titers stored at RT or 37 °C were compared to liquid control vaccine (stored at −80 °C). RRV-TV vaccine films were evaluated out to three months at varying storage temperatures, ranging from RT, 37 °C, 45 °C, and 50 °C.

Samples were tested using immunoperoxidase focus forming assay on MA104 cells on 96-well plates (Nest Scientific, Beltsville, MD, USA. TC-treated). PBV foams and vaccine films were reconstituted with serum-free Dulbecco’s modified Eagle medium (DMEM; Corning) and trypsinized (final trypsin concentration 5 µg/mL) for 1 h at 37 °C. Trypsinized samples were serially diluted with serum-free DMEM to 1 × 10^−8^ dilution. MA104 monolayers were washed with serum-free DMEM, inoculated with 50 µL of activated sample from each dilution and incubated at 37 °C for 1 h. After adsorption, 150 µL of DMEM (serum-free, 1 µg/mL trypsin) was added to each well, and incubated at 37 °C for 18 to 19 h. After incubation, cells were washed once with serum-free DMEM, then fixed in cold (−20°C) 80% acetone for 15 to 20 min at −20 °C, and air dried at room temperature. PBS + 0.5% Tween−20 was added to each well to wet the cell monolayer, then immediately aspirated. Cells were treated with goat anti-rotavirus antibody (Sigma Aldrich, St. Louis, MO, USA. AB1129; diluted 1:1000 in PBS + 1% BSA) and incubated at 37 °C for 1 h. Cells were washed three times with PBS + 0.5% Tween−20, then treated with HRP-conjugated rabbit anti-goat IgG antibody (Sigma Aldrich, St. Louis, MO, USA. AP106P; diluted 1:1000 in PBS + 1% BSA) and incubated at 37 °C for 1 h. Cells were washed three times with PBS + 0.5% Tween−20, then developed using an AEC staining kit (Sigma Aldrich, St. Louis, MO, USA. AEC101) according to kit instructions. Sodium azide (0.05% in PBS) was added to each well following development. Focus forming units were counted under an inverted microscope at 100× magnification.

### 2.8. Statistical Analysis

Pigs were randomly assigned into treatment groups upon derivation by animal care technicians, regardless of gender and body weight. Kruskal-Wallis test followed by Dunn’s test for multiple comparisons was used to evaluate cumulative diarrhea scores, days with diarrhea, area under the curve (AUC) of diarrhea, shedding onset day by ELISA and CCIF, and days with shedding by ELISA and CCIF. AUC of CCIF titers, mean peak titers and mean duration of diarrhea were analyzed by ordinary one-way analysis of variance (ANOVA). CCIF titers, antigen ELISA optical density (OD) values, and HRV-specific IgA, IgG, and VN antibody titers among the treatment groups were analyzed by two-way ANOVA with repeated measures and followed by Tukey’s test for multiple comparisons. Diarrhea scores of individual pigs were analyzed by Friedman’s test. All analyses were carried out using GraphPad Prism 8.0 (GraphPad Software, San Diego, CA, USA). Data of vaccine stability after storage was analyzed by two-way ANOVA using SigmaPlot 14.5 (Systat Software Inc., San Jose, CA, USA). For all analyses, a *p* value lower than 0.05 was accepted as statistically significant.

## 3. Results

### 3.1. Preserved Thermostable Film Vaccine Conffered Strong Protection against Diarrhea and Virus Shedding upon Challenge with VirHRV

**Figure 1:**
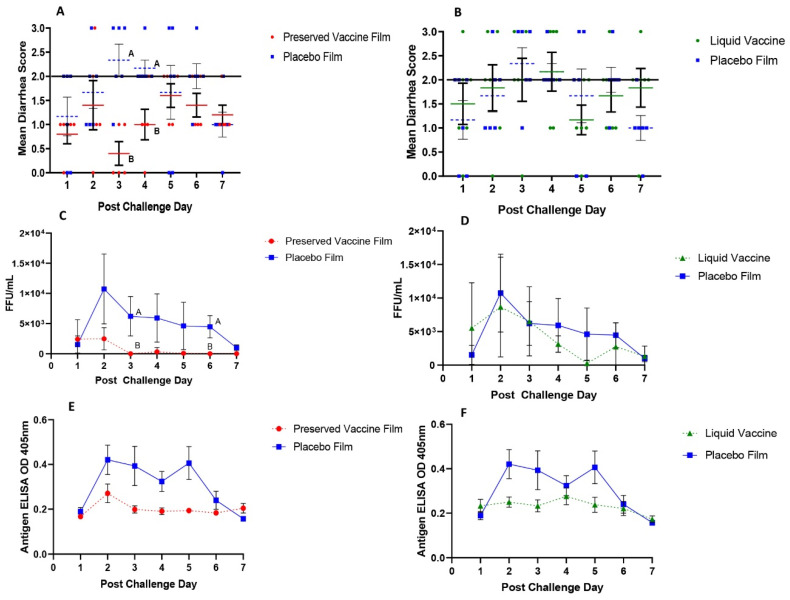
Diarrhea score and virus shedding postchallenge in Gn pigs vaccinated with preserved film vaccine (**A**,**C**,**E**) or liquid vaccine (**B**,**D**,**F**). The pigs vaccinated with preserved film vaccine had significantly reduced diarrhea on PCDs 3–4 compared to the placebo film group (**A**). Pigs in the preserved film vaccine group also showed a reduced overall virus shedding by CCIF (**C**) and antigen ELISA (**E**). The mean ± SEM bars of pig groups are indicated by solid horizontal line and vertical thicker lines, respectively (**A**,**B**). Fecal rotavirus antigen was measured by ELISA and results are expressed as OD units, and infectious virus particles were measured by CCIF and expressed as FFU/mL. Fecal samples from mock-infected pigs were used as negative controls in both antigen ELISA and CCIF. Different capital letters (**A**,**B**) indicate significant differences (*p* < 0.05) between groups at the same time point, while shared letters or no letters indicate no significant difference. ELISA, enzyme-linked immunosorbent assay; FFU, focus forming units; OD, optical density.

Vaccinated and control Gn pigs were challenged with VirHRV at PID 28 and were monitored daily for clinical signs of infection (diarrhea) and virus shedding from PCD 1 to PCD 7. For diarrhea, although the preserved film vaccine did not totally prevent diarrhea (four pigs had mild diarrhea for 1–2 days), pigs that received the preserved film vaccine had a significantly delayed onset of diarrhea (4.4 days), compared to the liquid vaccine (1.7 days) and the placebo film (1.8 days) vaccinated groups, as well as a significantly reduced mean diarrhea score on PCDs 3 and 4 (Table 1 and Figure 1A). Preserved film-vaccinated pigs had a significantly reduced mean duration of diarrhea (1.8 days) compared to the liquid vaccine (4.5 days) and placebo film vaccine (4.3 days) pig groups (Table 1). The preserved film vaccine significantly reduced the AUC of diarrhea compared to the liquid vaccine and placebo groups (6.8, 9.7, and 10.6, respectively) (Table 1). There were no significant differences in any parameters assessing the severity of diarrhea between liquid vaccine and placebo groups (Table 1 and Figure 1B).

For virus shedding, although rotavirus was detected by CCIF in the fecal samples of all the VirHRV-challenged pigs, the preserved film-vaccinated pigs only shed virus on the first two days and had significantly lower virus shedding titers by CCIF on PCDs 3, 6, and 7, whereas the liquid vaccine did not reduce virus shedding when compared to the placebo film controls (Figure 1C,D). Preserved film-vaccinated pigs showed significantly reduced duration of virus shedding (2.2, 5.7, and 6.8 days, respectively), mean peak titers (~4-fold lower) and AUC (~6- to 8-fold lower) of virus shedding compared to both placebo and liquid vaccine groups (Table 1). The amount of rotavirus antigen in fecal samples measured by ELISA OD values were substantially lower in both preserved film vaccine and the liquid vaccine groups compared to the placebo control from PCDs 2–6, but there were no significant differences on any time point (Figure 1E,F).

### 3.2. Strong Serum IgG and IgA and Interstinal IgA Antibody Responses were Induced by Both the Preserved Film Vaccine and the Liquid Vaccine

**Figure 2:**
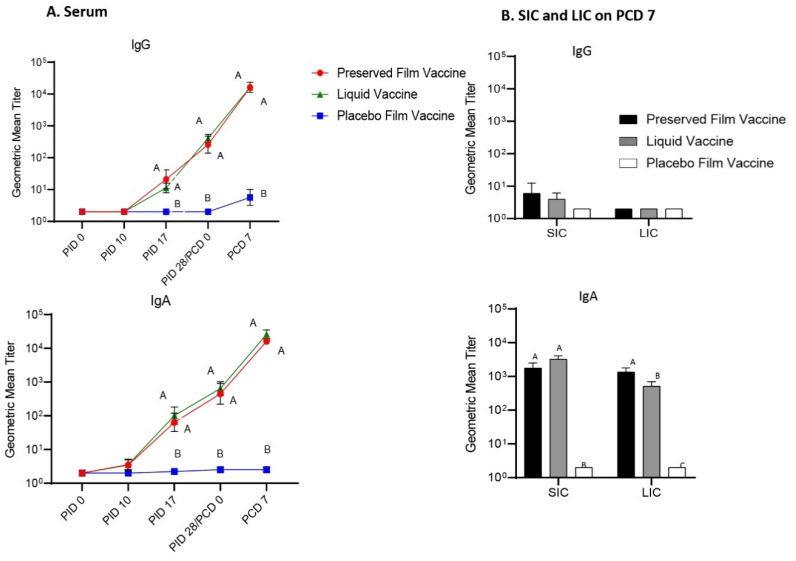
Geometric mean titers of Wa HRV-specific IgG and IgA antibodies in Gn pig serum samples collected at PID 0, PID 10, PID 17, PID 28, and PCD 7 (**A**), and in small intestinal contents (SIC) and large intestinal contents (LIC) collected on PCD 7 (**B**). Serum, SIC, and LIC samples were tested at a series of 4-fold dilutions, beginning at 1:4. All negative samples were given a titer of 2 to allow for data analysis and use in graphical depictions. Different capital letters (A, B, C) indicate significant differences (*p* < 0.05) between groups, while shared letters indicate no significant difference. Data points without capital letters indicate no significant difference between any of the groups at that time point.

Serum was collected from Gn pigs at PID 0, PID 10, PID 17, PID 28 (PCD 0), and PCD 7, as well as SIC and LIC on PCD 7 to evaluate antibody responses induced by the vaccination. Pigs vaccinated with both preserved film vaccine and liquid vaccine showed significantly higher serum IgA and IgG titers than placebo film-vaccinated pigs from PID 17 onward (Figure 2A). They both also had significantly higher IgA titers in SIC and LIC on PCD 7 compared to pigs in the placebo film group. Notably, however, IgA titers in the LIC of preserved vaccine group were significantly higher than the liquid vaccine group, and this is associated with the significantly higher protection conferred by the preserved vaccine (Figure 2B). Intestinal IgG titers were low and there was no difference in both SIC and LIC of any groups (Figure 2B).

### 3.3. Significantly Higher Titers of G1, G3, and G4 Rotavirus Serum VN Antibody Responses were Induced by the Preserved Film Vaccine Than the Liquid Vaccine at PCD 7

**Figure 3:**
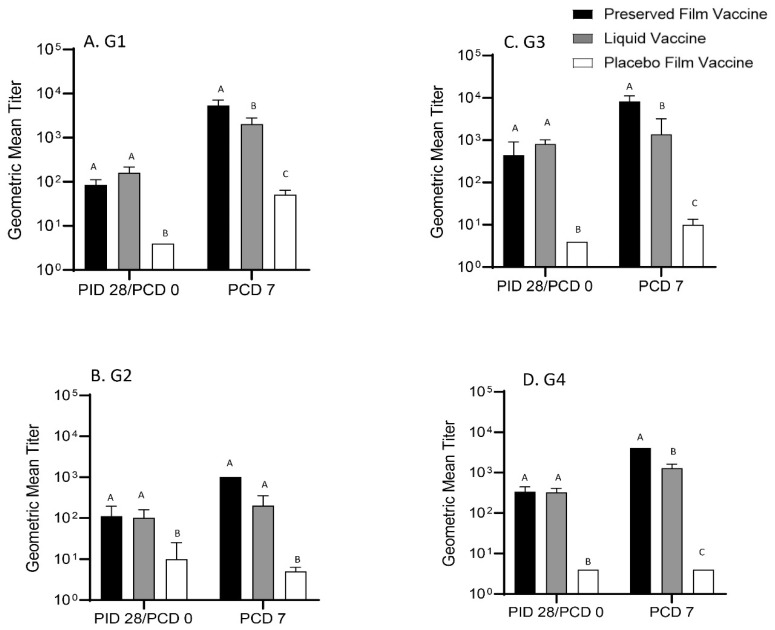
Geometric mean titers of virus-neutralizing (VN) antibodies in Gn pig serum samples at PID 28 and PCD 7. The sera were tested in a series of 4-fold dilutions, beginning at 1:4 in EMEM to 1:16384 for neutralization of 4 × 10^3^ virions of G1 (**A**), G2 (**B**), G4 (**D**) human-rhesus reassortant rotaviruses and G3 (**C**) RRV. Different capital letters (A, B, C) on the top of the bars indicate a significant difference (*p* < 0.05) between groups, while shared letters indicate no significant difference.

G1 to G4 rotavirus-specific VN antibodies were measured in serum samples from pigs at PID 28 (PCD 0) and PCD 7. The preserved film-vaccinated pigs had similar VN GMTs to the liquid vaccinated pigs at PID 28, but higher or significantly higher G1, G2, G3, and G4 VN antibody levels compared to the liquid vaccinated pigs on PCD 7 (Figure 3). This coincided with the significantly higher IgA titers in the LIC at PCD 7 and is associated with the significantly higher protection against diarrhea and virus shedding in the preserved film vaccine group.

In the preserved film vaccine group, the highest VN antibody GMT was against G3 at PID 28. The VN GMTs increased substantially from PID 28 to PCD 7 with the highest fold increase against G1 (52-fold), reflecting the anamnestic response to the homologous G1 rotavirus challenge, followed by G3 (18-fold), G4 (12-fold), and G2 (9-fold) rotaviruses (Table 2). In the liquid vaccine group, the highest VN GMT at PID 28 was also against G3. However, from PID 28 to PCD 7, it only increased 13-fold against G1, the same fold increase to G1 as in the placebo film group. Postchallenge, the VN GMT to G2, G3, and G4 rotaviruses only increased slightly (2–4 fold) in the liquid vaccine group and placebo film group (3-fold to G3 and no increases to G2 and G4).

### 3.4. Safety of Preserved Film and Liquid Vaccine in Neonatal Gn Pigs

Overall health status of the Gn pigs was monitored daily throughout the experiment by certified animal care staff. There was no intussusception after vaccination or VirHRV challenge in any of the pigs. There were no other signs of adverse effects after vaccination except occasional incidences of diarrhea (fecal scores ≥ 2) were recorded in the preserved film vaccine group (10), liquid vaccine group (8), and the placebo film group (3) after PID 10 (Supplemental Table S1). We changed the pigs’ diet from whole cow’s milk on PID 21 to Similac^®^ baby formula until the end of the study. Usually, the mild diarrhea caused by indigestion of cow’s milk can be resolved by switching to baby formula which contains less fat. On PID 28/PCD 0, there were still 1–2 pigs in each group that had diarrhea. Those diarrhea scores before challenge were taken into consideration as the covariance in a Kruskal-Wallis test, which could reduce the impact of the pigs who had diarrhea before challenge and could be potentially prolonged into postchallenge period. The analysis results showed there was no difference among treatment before challenge, and the results also showed that the prechallenge diarrhea scores only have a very little influence on the diarrhea scores after challenge.

### 3.5. Preservation by Vaporization Stabilizes Live Oral Rotavirus Vaccine at Ambient and High Temperatures

**Figure 4:**
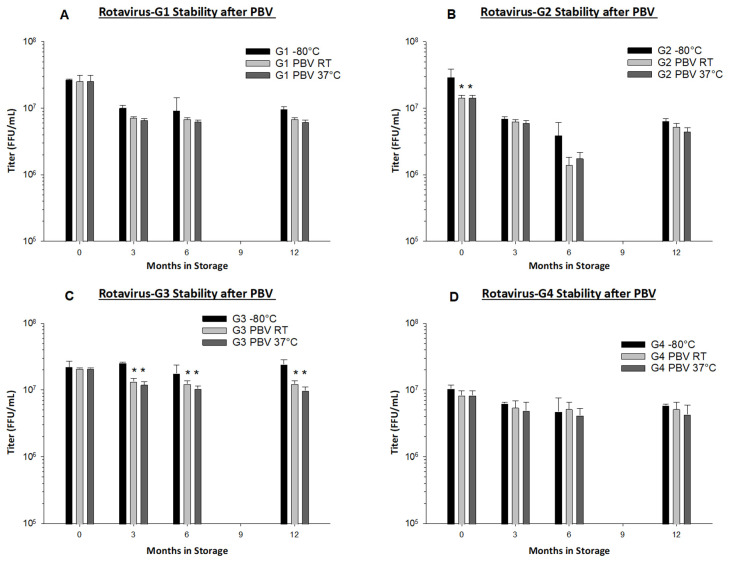
Long-term stability of preservation by vaporization (PBV) live rotavirus vaccines G1 (**A**), G2 (**B**), G3 (**C**) and G4 (**D**) at different temperatures was tested via immunoperoxidase focus-forming assay on MA104 cells. PBV provided high long-term stability and viral recovery for individual rotavirus vaccine strains after 1 year of storage at room temperature (RT) and 37 °C in the dry carbohydrate foam format. Samples of PBV vaccines stored at RT (22 ± 3 °C) and 37 °C were tested at initial yield (0 month), three months, six months, and 12 months in comparison to liquid control vaccine stored at −80 °C. Asterisk (*) indicates significance (*p* < 0.05) between test group and −80 °C control (two-way ANOVA). Less than 0.5 logs loss in measured titer was observed in all groups in comparison to −80 °C control vaccine, even groups where significance was noted.

After storage at different temperatures, live rotavirus vaccine titer was measured via immunoperoxidase focus forming assay. In carbohydrate foam format, no significant loss (determined as 0.5 logs or more of loss as compared to liquid control vaccine stored at –80 °C) was observed in any of the rotavirus serotype vaccines even after a 12-month storage period at 37 °C (Figure 4). Highest observed titer loss was seen in rotavirus serotype G3 after 12 months at 37 °C, measuring 0.39 logs of loss compared to frozen liquid control (Figure 4C).

Stability of RRV-TV vaccine films was also evaluated out to three months at varying storage temperatures, ranging from RT (22 ± 3 °C), 37 °C, 45 °C, and 50 °C (Table 3). Less than 0.5 logs of loss was measured at each storage time/temperature group. The PBV method confers excellent thermostability to live attenuated rotavirus vaccines in foam format and in films incorporating micronized PBV powder.

## 4. Discussion

Preservation by Vaporization is a very gentle and quick drying process. Primary drying only requires approximately 2 h, and less than 0.1 logs of titer loss occurs due to the drying process in all rotavirus serotypes with the exception of rotavirus G2, in which we observed 0.3 logs of loss (Figure 4B). From our long-term stability evaluation, we observed <0.5 logs of loss in PBV foams after drying and subsequent 12 months of storage at both RT and 37 °C in all serotypes (Figure 4). PBV-dried RRV-TV is also well-preserved during micronization and encapsulation in films, with <0.3 logs of loss observed. From the same film batch that was produced for use in Gn pigs, we observed no loss in titer during three months of storage at RT, and <0.2 logs loss at 37 °C (Table 3). We believe our dissolvable oral RRV-TV vaccine films to be thermostable and well-suited for cold chain-free storage, shipment, and delivery.

The immunogenicity and protective efficacy of the preserved thermostable dissolvable oral polymeric film RRV-TV vaccine were evaluated in neonatal Gn pigs in comparison with the reconstituted liquid RRV-TV vaccine. After two buccal doses of vaccine, the preserved film vaccine conferred significant protection against diarrhea and virus shedding upon challenge with the virulent Wa HRV. The preserved film vaccine significantly delayed the onset of diarrhea, shortened the duration of diarrhea, and reduced the AUC of diarrhea compared to the placebo film. The preserved film vaccine also significantly reduced virus shedding compared to the placebo film. Preserved film-vaccinated pigs had significantly reduced duration, mean peak titers, and AUC of virus shedding compared to pigs in the placebo film group. In contrast, the reconstituted liquid RRV-TV did not confer any protection. Among the various transmucosal vaccination routes, buccal mucosa is the best choice as it has excellent patient accessibility, can induce both humoral and cell-mediated protective immunity, especially strong mucosal immune responses [37], and the buccal area has direct access to the systemic circulation through the internal jugular vein [38]. It avoids the first pass effect leading to high bioavailability of the vaccine and can have a dose sparing effect. This may be another reason to explain the discrepancy between the similar immune responses but different protection conferred by the buccal preserved film and oral liquid vaccines.

The preserved film vaccine demonstrated high immunogenicity in neonatal Gn pigs. Two doses of the film vaccines induced strong IgG and IgA antibody responses in serum, LIC and SIC, and also high levels of serum VN antibody responses prechallenge to all four G types of the rotaviruses corresponding to the RRV-TV vaccine in this study. Postchallenge, the GMT of serum VN antibodies in preserved film vaccine group had the highest fold increases to all the four G types among the three treatment groups from prechallenge (Table 2), suggesting the preserved film vaccine primed for the strongest anamnestic responses against the VirHRV challenge. However, the preserved film vaccine did not totally prevent virus infection in any pigs. Virus shedding in the preserved film-vaccinated pigs was brief, mostly occurred at PCD 1–2. After PCD 2, there were only two pigs shedding low titers of rotavirus for one day at PCD 4 (CCIF titer 1600) and PCD 5 (400), respectively (Figure 1C). This type of protection is characteristic of T-cell mediated immunity. In previous studies of live oral HRV vaccines in Gn pigs, three doses of oral immunization with the Wa AttHRV vaccine prevented virus infection in 68% of the pigs upon challenge with the homologous Wa VirHRV [39], in which there was a significant correlation between intestinal and serum IgA antibody responses with the protection rate. It is perceivable that humoral immune responses did not play major role in the protective immunity conferred by the preserved film vaccine. The hallmark of T-cell mediated protection is that it does not totally prevent infection but the memory T cells induced by the vaccine residing in the small intestinal lamina propria can be reactivated quickly to secret anti-viral cytokines, become cytotoxic cells or facilitate anamnestic antibody responses to clear the virus infection postchallenge [40]. It is likely that the rotaviruses in the preserved film vaccine replicated significantly better in the pig intestine than the liquid vaccine and thus induced a much stronger memory T-cell response in these pigs, resulting in stronger protection against virus infection and diarrhea upon challenge with VirHRV. Unfortunately, T-cell responses were not measured in this study.

A possible reason for the different vaccine effectiveness between preserved film and liquid vaccine is the inclusion of antacid. The preserved film vaccine contains antacid in the film formulation, so it was simultaneously delivered to the pigs whereas liquid vaccine does not contain antacid. The sodium bicarbonate was fed to pigs 20 min prior to vaccine inoculation. It is possible that while the stomach pH may have been neutralized at first, within the 20 min timeframe, additional gastric acid sufficient to lower the pH was produced, enough to reduce the virus viability and hence vaccine efficacy.

Although not protective, the liquid vaccine induced similar magnitude of antibody responses as the preserved film vaccine in Gn pigs prechallenge. The serum IgG and IgA, and VN antibodies to all the four G type viruses, as well as IgA antibody responses in SIC induced by the liquid vaccine were comparable to the preserved film vaccine at PID 28. After the challenge, however, the VN GMTs in the liquid vaccine group was much lower than the preserved vaccine group because the fold increases were much smaller compared to the preserved film vaccine, indicating its weaker immunogenicity for memory responses.

In a large placebo-controlled efficacy trial of the RRV-TV in humans, no consistent relationship was found between the titers of any VN antibodies [Wa (G1), DS-1 (G2), P (G3), ST-3 (G4), RRV (G3)] or rotavirus-specific IgA following vaccination and protection against rotavirus, thus suggesting that serum antibody titers would not be useful markers of protection with the RRV-TV vaccine [41]. In our study, the Gn pigs vaccinated with the preserved film vaccine had significantly higher titers of IgA in LIC and significantly higher serum VN antibody titers to G1, G3, and G4 rotavirus on PCD 7 than the liquid vaccine, which may also indicate that the main immune effectors conferring the protection upon challenge were T cells. Stronger helper T-cell responses might have been primed by the preserved film vaccine, leading to stronger anamnestic antibody immune responses after infection caused by VirHRV challenge.

In addition to the stronger anamnestic intestinal and systemic immune responses induced by the preserved film vaccine, salivary IgA responses may have also played a role. Because the preserved film vaccine was administered buccally (but was not quickly dissolved), the result was a prolonged exposure to the mucosal surfaces in the mouth. It is possible that a virus-specific salivary IgA antibody response may have been stimulated by the buccal film vaccine, resulting in stronger protection against infection from oral virus challenge. Buccal mucosa is rich in immune cells such as dendritic cells and Langerhans cells and these cells are able to uptake vaccines in the film [38]. Rotavirus-specific antibody-producing B cells can then develop and reside in salivary glands or homing to intestinal mucosal surfaces through the common mucosal immune system and produce IgA antibodies [42]. Secondary IgA responses tend to be quicker, stronger and longer-lasting upon subsequent exposure to antigen than primary responses [43]. Since pigs were vaccinated twice, both with prolonged mucosal exposure to preserved film vaccine, we would expect to see an increased salivary IgA response after the second vaccination on PID 10, lasting long enough to mitigate oral challenge with VirHRV at PID 28. After natural infection by rotavirus or oral inoculation with RRV-TV in infants, ~50% of infants had rotavirus-specific saliva antibody responses; however the role of salivary IgA in rotavirus protective immunity was not studied [44,45]. Regrettably, we did not collect salivary samples from the pigs in this study. It will be interesting to measure salivary IgA antibody responses in future studies of animals or humans vaccinated with buccal film vaccines to validate this hypothesis.

The mucoadhesive dissolvable polymeric film vaccines are designed to adhere quickly once applied to the buccal mucosal membrane in the mouth and exposed to moisture. The film dissolves over time and releases the antacid ingredients and vaccine for oral delivery. Two initial safety studies were outsourced to determine toxicity of film ingredients and irritation/sensitivity on oral mucosal membranes. A preliminary 14-day toxicity study was performed by Charles River Laboratories using four groups of five male rats (20 in total), administered with either PBS solution, one sq. inch dose of film (full human dose), one-tenth sq. inch dose of film, or one sq. inch dose of film containing no PBV powder. Films used in the toxicity study contained no antacid; the CaCO_3_ antacid used is FDA-approved and fully characterized, and the excessive dose for rat size would have conflated any adverse clinical effects caused by high dose antacid with those caused by films. Doses were administered twice via oral gavage, on Days 1 and 7, and all groups were terminated on Day 14. Study results showed no clinical observations of toxicity, effects on body weight or food consumption, changes in hematology or clinical chemistry parameters, or effects on organ weight, and no macroscopic or microscopic findings at any dose level (unpublished data).

A preliminary oral mucosal irritation/sensitivity study in hamsters was performed by NAMSA. The right cheek pouch of 10 hamsters was implanted with full human dose film (containing both vaccine PBV powder and antacid). The left cheek pouch of all animals was implanted with polymethylmethacrylate (PMMA) disks which served as control article. Both articles were held in place with fitted collars. Good adhesion to hamster mucosal membrane was also observed after administration. Animals were euthanized after 24 h of exposure and pouch mucosa were subsequently removed for evaluation. The results showed no macroscopic or microscopic reactions of the RRV-TV film to hamster cheek tissue (unpublished data).

Due to the relative newness of the technology used to develop the buccal film, it was apparent during animal experiments that the formulation needs further improvement to offer a usable oral vaccine alternative in human infants. As mentioned above, the film comprised a high percentage of powders, causing it to be brittle, thick, and difficult to manipulate. Plasticity of the films was evaluated by observation of film behavior after repeated bending and folding. It was determined that film plasticity strongly decreases when the amount of total powders incorporated within a one sq. inch film increases above 60 mg. Due to dosing requirements, total mass of powder in our vaccine formulation was 120 mg, and the resulting films expressed brittle behavior, which made them difficult to manipulate and administer. To increase plasticity of the films, the mass of the vaccine powder per film can be substantially decreased by concentrating the viruses before drying. There is also significant room to reduce the mass of antacid per film without negatively affecting vaccine potency. A recent study [46] has demonstrated that 10 mL of 200 mM buffer concentration is sufficient to neutralize 4 mL of 0.1 N HCl, which mimics infant stomach conditions. To eliminate the negative effect of low gastric pH on orally delivered rotavirus vaccines, a dose of 40 mg of CaCO_3_ per film should be sufficient. Another study found no statistical difference in IgA seroconversion rate between Rotarix^®^ RIX4414 vaccine reconstituted with liquid CaCO_3_ buffer or with water [47]. The mass of CaCO_3_ used per film could be potentially lowered several times while still guaranteeing vaccine efficacy.

A formulation with a thinner, more flexible texture and quicker to dissolve film would be ideal as has been discussed in the review article by Uddin et al. [39]. Promisingly, this issue can be easily resolved by concentrating the starting bulk liquid vaccine, thus reducing the total mass of micronized vaccine powder that must be incorporated into the film. Future formulations will include more concentrated vaccine powder and lower amounts of antacid per film, resulting in thinner and more flexible films. Starting with bulk liquid rotavirus vaccine with 5–10 times higher concentration will allow production of adequately concentrated vaccine powders for this purpose.

Certainly, further investigations to identify the specific immune responses that mediated the strong protection by the preserved thermostable film vaccine are warranted. Investigating T-cell responses, including intestinal IFN-γ producing effector/memory T cells [48] and T follicular helper cells which play a critical role in helping B cells to produce antibody against foreign pathogens, would generate the insight on the cell-mediated immunity conferred by the vaccine [49]. Due to the novel route of administration, evaluating salivary antibody responses would further clarify mechanisms of protection, as well.

## 5. Conclusions

Our study demonstrated the safety, immunogenicity, and effectiveness of the thermostable, dissolvable oral film RRV-TV rotavirus vaccine using the neonatal Gn pig model of HRV infection and diarrhea. Two doses of the preserved film vaccine were highly immunogenic and conferred significant protection against virus shedding and diarrhea upon challenge with a high dose of a virulent G1 HRV. Associated with the strong protection, higher or significantly higher GMTs of anamnestic serum VN antibodies against each of the four serotype rotaviruses and significantly higher titers of G1 HRV-specific large intestinal IgA antibodies were induced by the preserved film vaccine compared to the non-protective liquid vaccine, implying strong memory immune responses (i.e., helper T-cell responses) were primed by the preserved film vaccine. The characteristics of the protection against virus infection suggest that T-cell mediated immunity played a more important role than humoral immunity in the preserved film-vaccinated pigs. Future studies of the vaccine should evaluate the correlation of effector/memory T-cell responses and protection. Our results provided solid evidence supporting the further film formulation optimization and evaluation of the thermostable, dissolvable polymeric oral film RRV-TV rotavirus vaccine in human clinical trials, which will lead to a licensed thermostable RRV-TV rotavirus vaccine that is of low cost in manufacture, storage, and shipment and is highly immunogenic and protective for neonates in the developing world.

## Figures and Tables

**Table 1:** Diarrhea and virus shedding in preserved film-vaccinated and control pigs after challenge with virulent Wa HRV.

		Clinical Signs of Diarrhea					Virus Shedding				
Treatments	*n*	% with Diarrhea	Mean Days to Onset	Mean Duration Days	Mean Cumulative Fecal Score	AUC of Diarrhea	% Shedding Virus	Mean Days to Onset	Mean Duration Days	Mean Peak Titer (FFU/g of feces)	AUC of Virus Shedding
Preserved Film	5	80% (4/5)	4.4 (1.12) ^A^	1.8 (0.49) ^B^	9.0 (0.89)	6.8 (1.14) ^B^	100% (5/5)	1.0 (0.25)	2.2 (0.45) ^B^	3120 (1242) ^B^	4080 (1418) ^B^
Placebo Film	6	100% (6/6)	1.8 (0.40) ^B^	4.5 (0.72) ^A^	12.0 (1.15)	10.6 (0.31) ^A^	100% (6/6)	1.0 (0.17)	6.8 (0.17) ^A^	12600 (1217) ^A^	33200 (3265) ^A^
Liquid Vaccine	6	100% (6/6)	1.7 (0.33) ^B^	4.3 (0.67) ^A^	13.3 (1.69)	9.7 (1.99) ^AB^	100% (6/6)	1.0 (0)	5.7 (0.61) ^A^	12533 (3023) ^A^	24767 (5819) ^A^

Note: a. Pigs were immunized twice with preserved film, placebo film buccally, or liquid vaccine orally at 5 (post-inoculation day [PID] 0) and 15 days (PID 10) of age. On PID 28, all pigs were orally challenged with 6 × 10^5^ FFU of virulent Wa HRV and monitored for diarrhea and virus shedding for 7 days postchallenge. b. Fecal consistency scores were used to assess diarrhea; scores are defined as 0: solid, 1: pasty, 2: semi-liquid, and 3: liquid. Scores of 2 or higher are considered diarrheic. Rotavirus shedding titers were determined by rotavirus antigen ELISA (detect viral antigen) and CCIF (determine the number of infectious viral particles). If there is no diarrhea or virus shedding, the mean days to onset were assigned as one day after the pigs were euthanized (8) for statistical analysis. c. Different letters indicate significant differences between groups (*n* = 5–6; *p* < 0.05), while shared letters or no letters indicate no significant difference. d. Numbers in parentheses are the standard error of the mean (SEM).

**Table 2:** Serum VN antibody GMT increases from prechallenge (PID 28) to postchallenge (PCD 7).

GMT	PID 28	PCD 7	Fold Increase	PID 28	PCD 7	Fold Increase	PID 28	PCD 7	Fold Increase	PID 28	PCD 7	Fold Increase
Group/type	**G1**			**G2**			**G3**			**G4**		
Preserved film vaccine	111	5793	52	111	1024	9	446	8192	18	338	4096	12
Liquid vaccine	161	2048	13	102	203	2	813	1380	2	323	1290	4
Placebo film vaccine	4	51	13	4	5	1	4	10	3	4	4	1

**Table 3:** Stability of PBV RRV-TV dissolvable films.

Storage Time	Storage Temperature	Mean Film Titer (FFU)	Stdev	Logs of Loss
**0 month**	--	5.80 × 10^5^	±0.27 × 10^5^	−0.24
**2 weeks**	50 °C	4.01 × 10^5^	±0.43 × 10^5^	−0.40
**1 month**	RT	5.73 × 10^5^	±0.20 × 10^5^	−0.24
	37 °C	4.89 × 10^5^	±0.11 × 10^5^	−0.31
**2 months**	RT	5.42 × 10^5^	±0.09 × 10^5^	−0.27
	45 °C	3.57 × 10^5^	±0.12 × 10^5^	−0.45
**3 months**	RT	5.54 × 10^5^	±0.16 × 10^5^	−0.26
	37 °C	4.01 × 10^5^	±0.27 × 10^5^	−0.40

Note: Logs of loss calculated from target titer of 1 × 10^6^ FFU per film tested.

## Data Availability

All the data supporting the reported results can be found in this paper.

## Supplementary Materials

The following are available online at https://www.mdpi.com/article/10.3390/vaccines9050437/s1, Table S1: Diarrhea incidences in Gn pigs from PID 0 to PID 28/PCD 0 before challenge.
