# Supplementary material for: Thermostable, Dissolvable Buccal Film Rotavirus Vaccine Is Highly Effective in Neonatal Gnotobiotic Pig Challenge Model
Source: Vaccines (Basel). 2021 Apr 30;9(5):437. doi: 10.3390/vaccines9050437 (PMC8147248; doi:10.3390/vaccines9050437)
Supplement: Supplementary file 1 [file vaccines-09-00437-s001.zip › vaccines-1183161-supplementary.pdf]

Table S1. Diarrhea incidences in Gn pigs from PID 0 to PID 28/PCD 0 before challenge[a]

Group	Placebo film						Liquid vaccine						Preserved film vaccine				
PID/PCD	Pig 1	Pig 2	Pig 3	Pig 4	Pig 5	Pig 6	Pig 1	Pig 2	Pig 3	Pig 4	Pig 5	Pig 6	Pig 1	Pig 2	Pig 3	Pig 4	Pig 5
0																	
10							2	2							2		
16							2	2									
17		2					3			2			2	2	2		
23													2	2	2		2
24		2															
28/0			3				2	3					2				2

	Placebo film	Liquid vaccine	Preserved film vaccine
Incidence of diarrhea	3	8	10[b]
Total diarrhea score from PID 0-28	6	19	20

Note:

a. Pigs were monitored for overall health status and especially for diarrhea by trained staff. When there is a sign of diarrhea, rectal swabs were taken and fecal consistency scores were recorded. Scores of 2 or higher are considered diarrheic. Blank cells in the table indicate there is no diarrhea in the pigs. Total diarrhea score is the sum of the scores in each group from PID 0-28.

b. The incidence of diarrhea in the preserved film vaccine group was significantly higher than the placebo film group from PID 0 to PID 28/PCD 0 (p=0.0124; Chi-Square Pearson test).
